# Burden of malaria in mobile populations in the Greater Accra region, Ghana: a cross- sectional study

**DOI:** 10.1186/s12936-017-1751-x

**Published:** 2017-03-09

**Authors:** Nouhoum Diallo, Patricia Akweongo, Ernest Maya, Moses Aikins, Bismark Sarfo

**Affiliations:** 1 School of Public Health, University of Ghana, Legon-Accra, Accra, Ghana; 2 Malaria Research and Training Center/Department of Epidemiology and Infectious Diseases/University of Science Techniques and Technologies of Bamako, Bamako, Mali

## Abstract

**Background:**

The burden of malaria in mobile populations remains poorly documented in sub-Saharan Africa. This study determined the prevalence of malaria among hawkers and long-distance truck drivers in the Greater Accra region of Ghana.

**Methods:**

A cross-sectional design using consecutive sampling method between June and July 2016 in Accra and Tema in Ghana was used in this study. The study population was hawkers who roam and sleep in the Market Streets, and long-distance truck drivers. Participants completed closed ended interview questionnaires on socio-demographic characteristics, primary residence and knowledge about malaria. Rapid diagnostic test and thick blood smears of each participant were stained with Giemsa and read using microscopy. Geographical position system (GPS) was used to collect the station locations of these mobile populations.

**Result:**

The overall prevalence of malaria was 15.1% and *Plasmodium falciparum* was responsible for all malaria infection. The malaria prevalence was 18.9 and 10.9% respectively among hawkers and truck drivers (p < 0.05). The hawkers, the single and the no formal educated participants were more likely to get malaria than the long-distance truck drivers (OR = 1.91, 95% CI 1.07–3.42), the married (OR = 1.94 95% CI 1.11–3.40) and the educated participants (OR = 2.56 95% CI 1.10–5.93), respectively. After controlling for other variables, marital status (OR = 2.60 95% CI 1.43– 4.73) and educational level (OR = 2.70 95% CI 1.08–6.77) were statistically significantly associated with malaria.

**Conclusion:**

This study demonstrated that the prevalence of malaria is high among hawkers and long distance truck drivers. Sociodemographic characteristics, such as marital status, occupation and educational level are significantly associated with malaria. The station locations as determined by GPS technology will make these mobile populations easier to reach for any malaria intervention.

## Background

*Plasmodium falciparum* is the most important threat to public health at a global scale, responsible for more than 90% of the World’s malaria mortality [1]. An estimated 212 million new cases of malaria occurred worldwide in 2015 (range: 148–304 million) and the majority of the cases occurred in the African Region (90%). [2]. According to a World Health Organization (WHO) report, malaria is responsible for approximately 1800 admissions at health care system and 10 deaths for every 100,000 population in Ghana [3]. The movement of human populations has been described as a major challenge tackling malaria control and elimination programmes since they require a knowledge of how the spatial distribution of malaria shifts through time and across multiple locations that become interconnected through population displacements [4]. Mobile populations from higher transmission settings risks reintroduction and resurgence in malaria-free areas, and has compromised elimination efforts in the past years [5, 6]. In non-elimination areas, understanding the dynamics of parasite movements from local hotspots of transmission is crucial for the development of appropriate surveillance and response system by identifying both the regions where imported infections originate and how they may contribute substantially to transmission [7].

Ghana and many sub-Saharan African countries have been experiencing rapid population growth and urbanization with high rates of migration into the cities and large towns, creating unemployment and overcrowding [8]. Most people without accommodation move around in the cities, do casual work and sleep wherever they find space during the night. Mobile populations, such as hawkers and long-distance truck drivers are all among the malaria at-risk populations who do not benefit from protection against mosquitoes, such as long-lasting, insecticide-treated bed nets (LLIN). Different features make migrants an at-risk of malaria population, including their socio-cultural context, economic activities, accessibility to health services, access to anti-malarials, service availability and readiness, behavioural parameters, language barriers, emergence of drug-resistant strains, activities for social mobilization, ecology, access to safe water and sanitation [9].

These groups tend to carry malaria parasites and could be a major source of residual transmission that could thwart the effort of any control strategy. Understanding malaria transmission in these mobile populations will be crucial in developing targeted interventions by the National Malaria Control Programmes [10].

In the literature, intra-country mobility and malaria risk remain poorly documented in sub-Saharan Africa. The magnitude of malaria in mobile populations and its contribution to the local malaria situation in Ghana is unknown. This study assessed the burden of malaria in mobile populations and their malaria-related risk factors in the Greater Accra Region of Ghana. The outcome of this study provides very useful information that will bridge the knowledge gap for programmatic improvement of malaria control in Ghana and the sub-region.

## Methods

### Study area

The study was conducted in two cities, Accra and Tema in the Greater Accra Region, one of the country’s ten administrative regions. It covers an area of about 420 km^2^ [11], with an estimated population of 4010,054 by the Statistical Service census of 2010. In 2010, malaria positive test results from Accra Metropolis was 8% [12]. Madina is a particularly interesting setting to examine the relationship between migration and malaria prevalence. It has a large migrant community. Madina is a suburb of Accra, the capital of Ghana, and in the LA-NKwantanang-Madina Municipal Assembly, in the East district of the Greater Accra Region. It lies about seventeen kilometres to the northeast of Accra, about four kilometres beyond the University of Ghana campus.

Tema is located in the Greater Accra Region with an estimated population of 402,637, which accounts for 392,044 people from urban areas and 10,593 from rural areas according to the Ghana Statistical Service census of 2010. According to the Ghana Health Service, District Health Information Management System data from January to October 2012, the percentage of malaria positive tests was about 12% in Tema. Tema has a port, which is the bigger of the two seaports of Ghana, handling 80% of the country’s national exports and imports. The harbour is situated along the Gulf of Guinea, 18 miles from the capital Accra, and it serves both as a loading and unloading port for goods, both for Ghana and the land-locked countries to the north, such as Burkina Faso, Mali and Niger. Long-distance truck drivers are present in this city, and they convey goods from the harbour to other parts of Ghana, and neighbouring countries.

### Study population (mobile population)

Mobile populations are defined as non-residents of the study area who had travelled there within the previous weeks and without accommodation. Most of the hawkers had travelled from the Northern part of Ghana to Accra the capital city in search of job opportunities as head porters. They often move around the market areas and assist shoppers in carrying their goods to their vehicles for a fee.

The truck drivers in this study are those who use their trucks in carrying goods from Tema harbour to the Northern part of Ghana as well as those who carry goods to the Economic Community of West African States (ECOWAS) member countries such as Mali, Burkina Faso and Niger.

### Sampling procedure and data collection

It was estimated that the prevalence of malaria among mobile population (long-distance truck drivers) in Niger Delta of Nigeria was 35% [13]. Using the formula, n = (z^2^pq)/d^2^ with n = sample size, p = proportion of malaria in mobile population (35%); q = 1−p; z = 1.96 and d = 5% at 95% confidence interval sample size of 350 study participants was estimated for this study. The study team compiled initial information from officials at Ghana Ports and Harbours Authority and the Queen mother of Madina Market Women Association, to identify locations and times in the study area that hawkers and long-distance truck drivers could be contacted. Mobile populations was defined as a non-resident of the study area who had travelled there within the previous weeks and without accommodation. Participants were recruited within each location using a consecutive sampling technique between June and July 2016. Volunteers were eligible if they were mobile populations (hawkers or long-distance truck drivers as defined above), aged 18 years or older, have the ability to provide informed consent and living in Madina market or Tema Harbour during the study period. Participants who consented were examined by a member of the study team who is a clinician. Subsequently, data on age, sex, education, knowledge of malaria transmission and prevention, and sleeping and mobility pattern were obtained through a closed ended questionnaire. Finger prick blood samples were also collected from participants to make thick smears, and examined for asexual parasites after staining with 5% Giemsa. Blood smears were read within 1 week after collection, by two trained microscopists using WHO standard procedures. Malaria parasitaemia was defined by the presence of asexual form of *Plasmodium* by microscopic examination. Asymptomatic malaria was defined as absence of history of fever and symptomatic malaria as presence of history of fever in the past 2 weeks. Participants were also screened with malaria Rapid Diagnostic Test using CareStart™ Malaria (Histidine Rich Protein 2) HRP2(Pf) (Access Bio, INC, 65 Clyde Road Suite A Somerset NJ 08873 USA) recommended by the Ghana National Malaria Control Programme. Positive cases with Rapid Diagnostic Tests were managed with artemether 20 mg+ lumefantrine 120 mg. The location and distribution point of these mobile populations were collected using Geographical Position System technology (GPSMAP^®^ 62, GARMIN, Kansas city, USA).

### Quality assurance and quality control

Parasite density was estimated by counting the number of asexual parasites per 200 leukocytes and multiplying by 40, assuming 8000 leukocytes/mL. Microscopists were kept blinded from the RDTs result until the smear parasite count was provided. Double entry for all data were reported.

### Data processing and analysis

Data were double entered using Microsoft Excel. Statistical analyses were performed using Statistical Package Stata, version 13.0 (StataCorp LP. College Station, Texas, USA). Frequency and percentage were presented for categorical variables. Malaria test was categories into two: No malaria (absence of malaria parasites in the blood analysed by microscopy) and malaria (presence of malaria parasites in the blood analysed by microscopy). Chi square or fisher’s exact test were used to assess the differences in sociodemographic and malaria knowledge factors between subjects. Subsequently, simple and multivariable logistic regression models were employed to analyse the risk factors of malaria. Both unadjusted and adjusted logistic regression models were performed. Odds ratios (OR) with 95% confidence intervals (95%, CI) were presented. Two tailed p values <0.05 were considered statistically significant.

Data from the Geographical Position System (GPS) were managed with a software called Base camp, produced by Environmental System Research Institute (ESRI, Kansas City, USA). Base camp is specifically designed for Garmin GPS’s. Data were first downloaded in Gpx format. Gpx is a format that Microsoft excel readily accepts for data or information analysis. The data could have easily been downloaded in “shapefile” format which is actually the format that would be required in the long run to make the analysis. Yet, the former was chosen to link each GPS point collected on the field to the exact questionnaire administered on the field of study. Since Gpx format readily accepts spreadsheet, it then makes it prudent and best to use that format to link both questionnaire information and GPS data.

After GPS data were collected and cleaned in the spreadsheet, a Geographical Information System (GIS) software was used for data management. Arcgis version 10.2.2 (ESRI, Kansas City, USA) was used in the data management procedure to generate the map showing sampling sites and malaria cases among the study participants.

### Ethical consideration

All study procedures were clearly explained to participants while obtaining informed consent. Study participants were assured of the confidentiality, data safety and appropriate data usage. The study received approval from the Ghana Health Service Ethics Committee (GHS-ERC 72/02/16).

## Results

### Sociodemographic characteristic of participants

Overall, 390 people aged 18–70 years old participated in the study. A total of 205 (52.56%) participants were from Madina market and the rest 185 (47.44%) from Tema Harbour. In all, 216 (55.38%) were males, females were 174 (44.62%) and 62.82% (245) were married. Participants were involved in two main occupation 206 (52.82%) were hawkers and long-distance truck drivers were 184 (47.18%). Regarding their educational status, 83 (21.28%) obtained at least secondary school education, 81 (20.77%) had primary school education and the majority 226 (57.95%) had no formal education (Table 1).

**Table 1:** Sociodemographic characteristics of participants

Variable	Frequency (N = 390)	Percentage (%)
Age, year		
<20	72	18.46
20–29	177	45.38
30–39	71	18.21
40–70	70	17.95
Locality		
Medina market	205	52.56
Tema harbour	185	47.44
Gender		
Male	216	55.38
Female	174	44.62
Marital status		
Single	145	37.18
Married	245	62.82
Occupation		
Truck driver	184	47.18
Hawker	206	52.82
Educational level		
No schooling	226	57.95
Primary	81	20.77
At least secondary	83	21.28

### Malaria prevalence and malaria knowledge factors

**Fig. 1:**
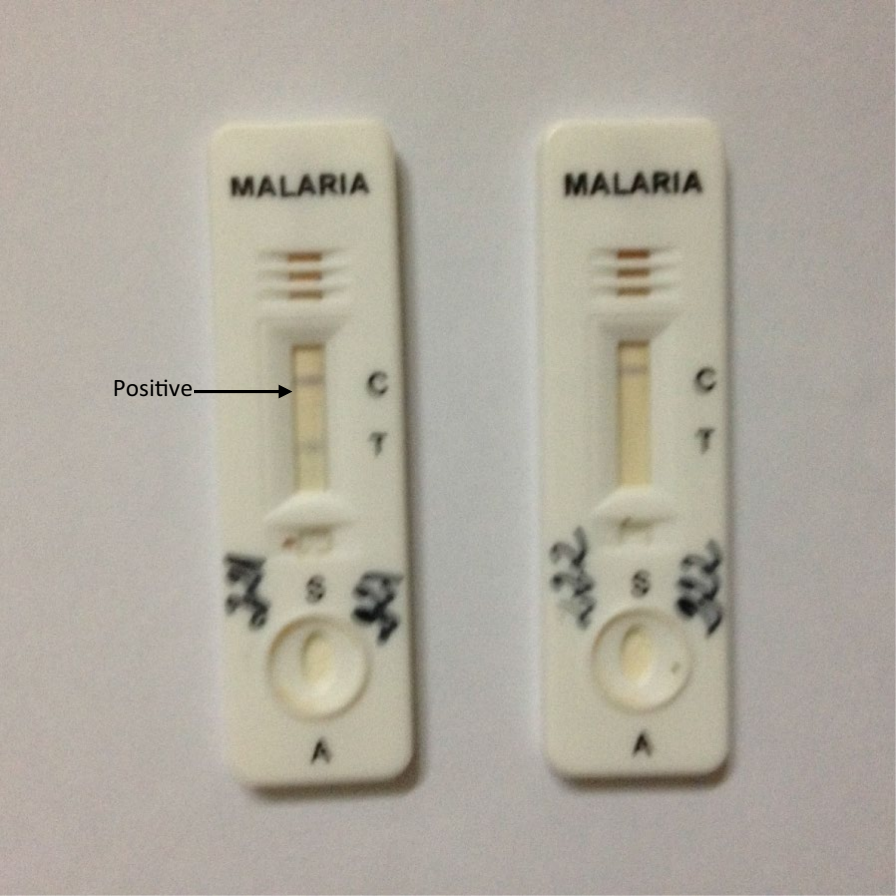
Rapid diagnostic test (RDT) results: positive (221) and negative (222). *C* control and *T* test result

The prevalence of malaria in this study was 15.13% using microscopy and 15.38% with rapid diagnostic tests (Fig. 1). *Plasmodium falciparum* was responsible for all malaria infection observed in this study. Most participants had history of fever in the past 2 weeks. 360 (92.31%) had no insecticide-treated bed net, while 30 (7.69%) had a bed net. A total of 365 (93.59%) knew about malaria, while 25 (6.41%) said they didn’t know. 85.90% (335) of the participants stated the correct mode of malaria transmission. Out of 390 participants, 332 (85.13%) stated correct measures of preventing malaria, while 58 (14.87%) said they did not know (Table 2).

**Table 2:** Malaria prevalence and malaria knowledge factors

Variable	Frequency (N = 390)	Percentage (%)
Microscopy result		
Negative	331	84.87
Positive	59	15.13
RDT result		
Negative	330	84.62
Positive	60	15.38
History of fever		
No	152	38.97
Yes	238	61.03
Have a bed net		
No	360	92.31
Yes	30	7.69
Know malaria		
No	25	6.41
Yes	365	93.59
Malaria transmission		
No	55	14.10
Yes	335	85.90
Prevention methods		
No	58	14.87
Yes	332	85.13

### Sociodemographic characteristics associated with malaria and its prevalence

The epidemiological characteristics of malaria and the sociodemographic characteristics are reported in Table 3. The Chi square or Fisher’s exact test showed that the prevalence of malaria among participants had significant differences (p < 0.05) in locality, marital status, occupation, educational level, while it had no significant difference (p > 0.05) in age groups and gender.

**Table 3:** Sociodemographic characteristics associated with malaria and its prevalence

Variable	No malaria	Malaria	p value
Age, year (n, %)			0.055
<20	57 (79.17)	15 (20.83)	
20–29	148 (83.62)	29 (16.38)	
30–39	60 (84.51)	11 (15.49)	
40–70	66 (94.29)	4 (5.71)	
Locality (n, %)			0.024*
Accra	166 (80.98)	39 (19.02)	
Tema	165 (89.19)	20 (10.81)	
Gender (n, %)			0.296
Male	187 (86.57)	29 (13.43)	
Female	144 (82.76)	30 (17.24)	
Marital status (n, %)			0.018*
Single	115 (79.31)	30 (20.69)	
Married	216 (88.16)	29 (11.84)	
Occupation (n, %)			0.027*
Truck driver	164 (89.13)	20 (10.87)	
Hawker	167 (81.07)	39 (18.93)	
Educational level (n, %)			0.019*
No schooling	182 (80.53)	44 (19.47)	
Primary	74 (91.36)	7 (8.64)	
At least secondary	75 (90.36)	8 (9.64)	

*No malaria* absence of malaria parasites in the blood analysed, *Malaria* presence of malaria parasites in the blood analysed; * p < 0.05

### Malaria knowledge factors associated with malaria and its prevalence

The epidemiological characteristics of malaria and malaria knowledge factors are reported in Table 4. The Chi square or fisher’s exact test showed that the prevalence of malaria among participants had no significant difference (p > 0.05) in history of fever in the last 2 weeks, have a bed net, know malaria, knowledge of malaria transmission and prevention methods.

**Table 4:** Malaria knowledge factors associated with malaria and its prevalence

Variable	No malaria	Malaria	p value
History of fever			0.147
No	124 (81.58)	28 (18.42)	
Yes	207 (86.97)	31(13.03)	
Have a bed net			0.065
No	302 (83.89)	58 (16.11)	
Yes	29 (96.67)	1 (3.33)	
Know malaria			0.482
No	20 (80.00)	5 (20.00)	
Yes	311 (85.21)	54 (14.79)	
Malaria transmission			0.277
No	44 (80.00)	11 (20.00)	
Yes	287 (85.67)	48 (14.33)	
Prevention methods			0.377
No	47 (81.03)	11 (18.97)	
Yes	284 (85.54)	48 (14.46)	

*No malaria* absence of malaria parasites in the blood analysed, *Malaria* presence of malaria parasites in the blood analysed

### Factors associated with malaria from logistic regression

All statistically significant variables in univariate analysis were applied to perform a multivariate logistic regression analysis, and the results are presented in Table 5. The results showed that marital status, occupation and educational level were significantly associated with malaria. The hawkers, the single and the no formal educated participants were more likely to get malaria than the long-distance truck drivers (OR = 1.91, 95% CI 1.07–3.42), the married (OR = 1.94, 95% CI 1.11–3.40) and the educated participants (OR = 2.56 95% CI 1.10–5.93), respectively. After controlling for occupation marital status (OR = 2.60, 95% CI 1.43–4.73) and educational level (OR = 2.70, 95% CI 1.08–6.77) were statistically significantly associated with malaria.

**Table 5:** Factors associated with malaria from logistic regression

Variable	OR^a^ (95% CI)	p value	OR^b^ (95% CI)	p value
Marital status				
Married	1		1	
Single	1.94 (1.11–3.40)	0.020*	2.60 (1.43–4.73)	0.002**
*Occupation*				
Truck driver	1		1	
Hawker	1.91 (1.07–3.42)	0.028*	1.74 (0.90–3.36)	0.099
*Educational level*				
Primary	1		1	
No schooling	2.56 (1.10–5.93)	0.029*	2.70 (1.08–6.77)	0.034*
At least secondary	1.13 (0.39–3.27)	0.825	1.20 (0.40–3.52)	0.741

*OR* Odds ratio, *95% CI* 95% confidence interval

* p < 0.05

** p < 0.01

^a^Crude OR

^b^Adjusted for all other variables included in the table

### Localizations of the halting point of this mobile populations using GPS technology

**Fig. 2:**
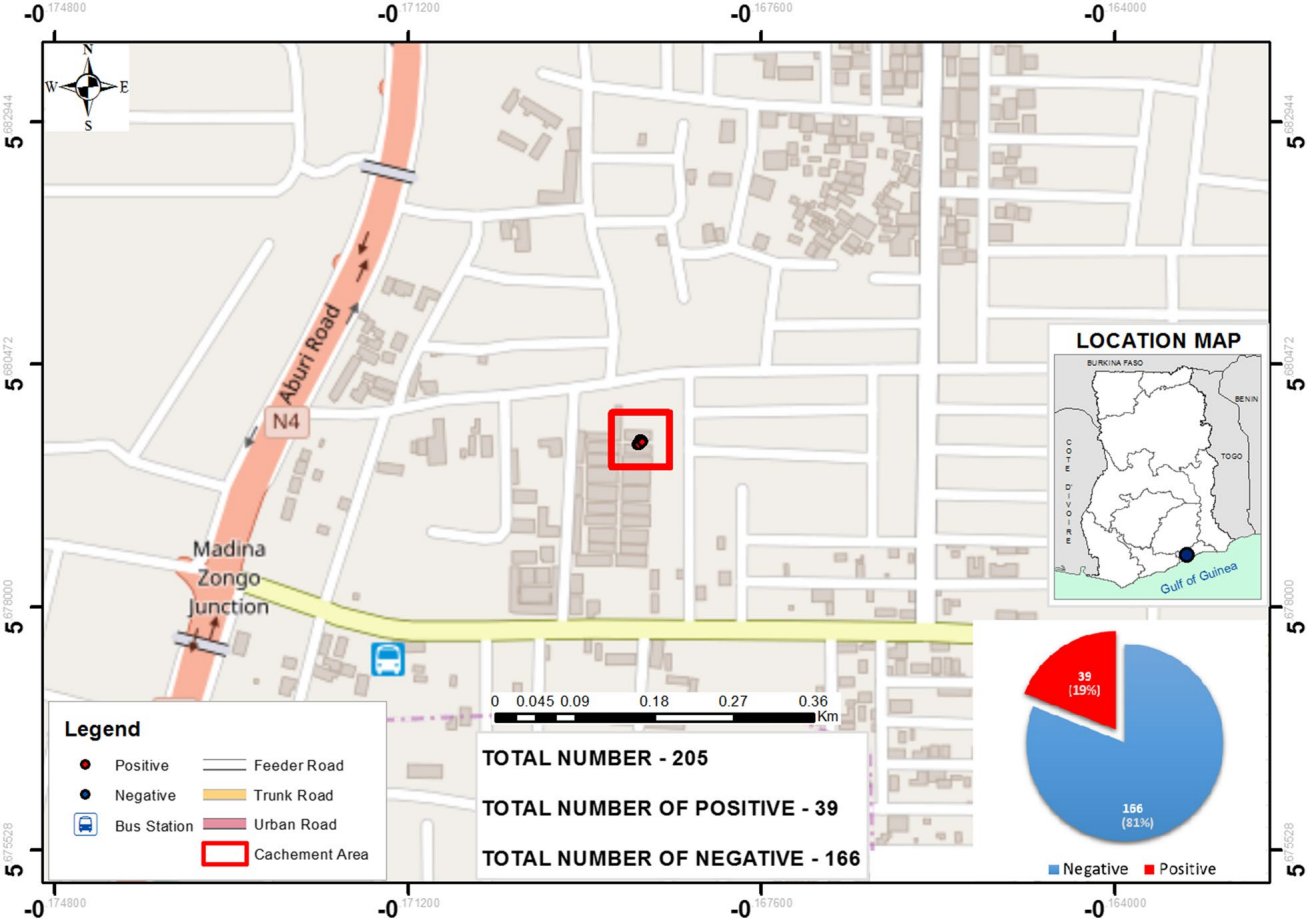
Map showing Madina market and malaria prevalence

**Fig. 3:**
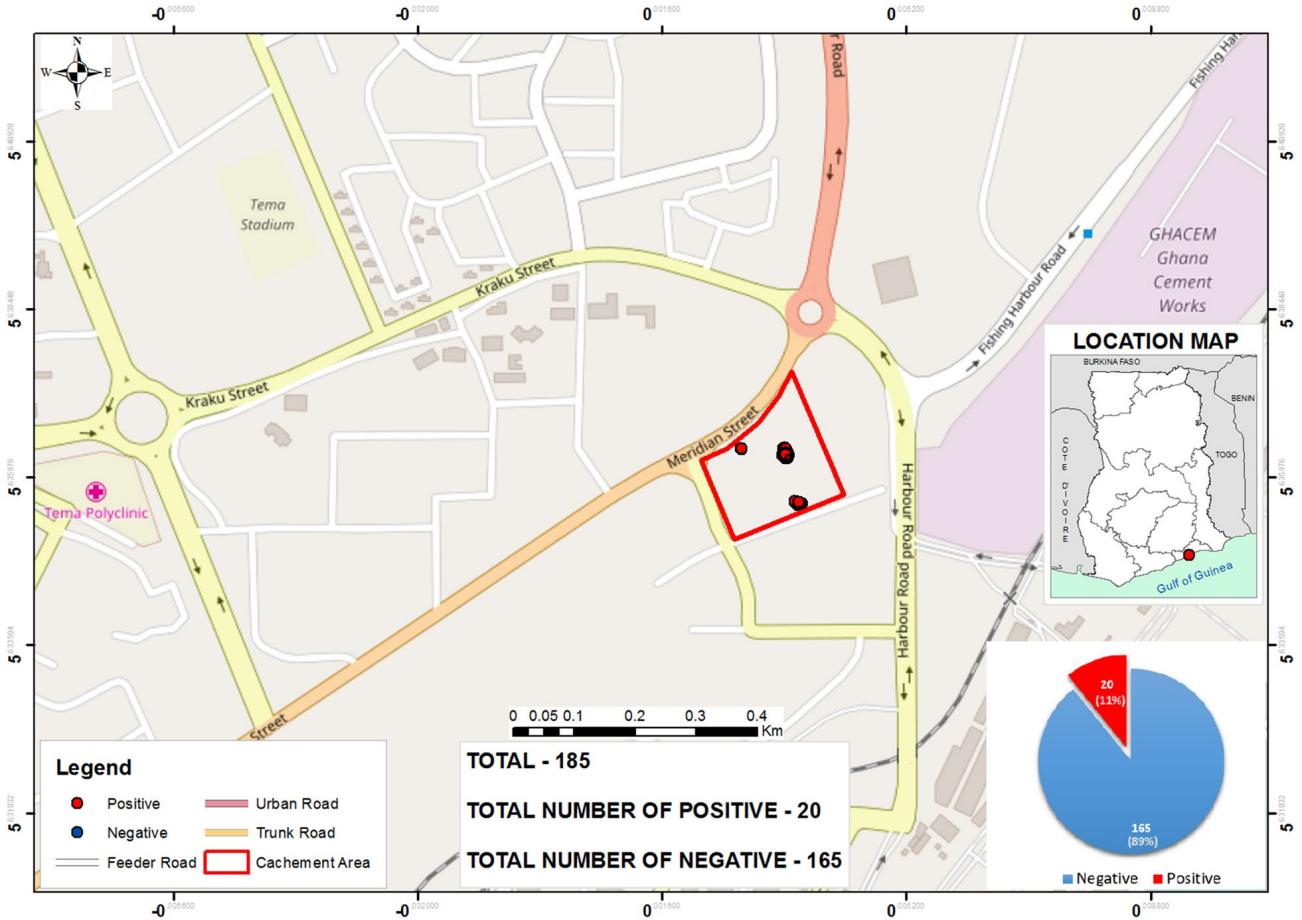
Map showing Tema harbour and malaria prevalence

The maps in Figs. 2 and 3 show the distribution of positive and negative malaria cases determined by microscopy at the Madina market and Tema harbour, respectively. The insert pie chart provides a clear-cut and distinct representation of the different proportions of malaria cases in the study site.

## Discussion

The microscopy remains the gold standard for malaria diagnosis. This study indicates that the prevalence of malaria was 15.1% in these mobile populations. This prevalence of malaria seems high in this adult populations from an area of malaria endemicity, where people develop immunity that protects them against high parasitaemia and clinical disease over a number of years [14]. However hawkers and long-distance truck drivers are among the malaria at-risk populations who do not benefit from some of the malaria intervention programs including the free distribution of LLINs that protect against mosquitoes bites and reduce malaria incidence [15]. The prevalence of malaria in our study is higher than a study conducted among migrant farm workers in Amhara region, in Ethiopia. The prevalence of malaria in this study using rapid diagnostic test was 12% of 605 participants [16]. The difference in the prevalence between the two studies could be explained by the proportion of participants who had access to LLIN. This proportion was 7.9% in the Greater Accra region, in Ghana and 11.9% in the Amhara region, in Ethiopia. This study prevalence was largely higher than an adult women clinical study conducted in Wakiso district of Uganda. This study found a prevalence of 2.3% among 86 participants [17]. The active case detection used in the study and the stable populations status of the non-pregnant adult women in Wakiso district, Uganda could justify this observed difference.

The study found a prevalence of 18.9 and 10.9%, respectively among hawkers and long-distance truck drivers indicating higher prevalence among hawkers than truck drivers. Hawkers move around in the market, and sleep wherever they find space during the night. Long-distance truck drivers on the other hand are very mobile and travel through Ghana ten administrative regions and neighbouring countries. Most of the drivers had to frequently stay overnight and regularly sleep in their trucks to protect their loads. Most (92.3%) of the study participants, had not used LLINs.

The study showed that asymptomatic malaria cases of 18.4% were higher than symptomatic cases of 13%. In malaria endemic area, adult populations develop immunity that protects against high parasitaemia and clinical disease after some years of continue exposition to mosquito bites [14]. A study conducted on malaria parasitaemia among long-distance truck drivers in the Niger delta of Nigeria reported a prevalence of 35%. The majority of the truck drivers studied were asymptomatic although some of them complained of regular fatigue, aches and weakness [13]. The reason for the high prevalence observed in the Nigeria study may be due to the fact that the study was carried out in an area of high incidence of malaria compare to Greater Accra region of Ghana. European visitors to the area described it as the “white man’s grave-yard” because of the high malaria related mortality rate they experienced [13].

The study showed that marital status, occupation and education level are risks factors strongly related to malaria. Those who are single had a higher odds of getting malaria than those who are married in this study. This may be due to the use of LLIN which is one of the strategies to reduce morbidity and mortality of malaria. The implementation of intervention that made LLIN available and free of charge especially for children and pregnant women could help married couple to be protected against mosquito bites. Marital status was a significant determinant of LLIN use in an urban area of Lagos state, Nigeria [18].

The study found that ‘no formal educated’ participants were more susceptible to malaria than ‘formal educated’ participants. This could be explained by the fact that those who have reached at least primary level of education might have been taught lessons on malaria in school, and are also more likely to read and comprehend malaria messages on tracts, radio or television. These findings were confirmed by others who reported that the knowledge of malaria was strongly associated with the level of formal education. Education remains a powerful tool that empowers people to enable them make decisions for themselves and influence their families [19].

The study revealed that majority (93.59%) of participants had heard about malaria before. This is not surprising as malaria is a major public health problem in Ghana and the sub-region. The National Malaria Control Programme (NMCP), under the Advocacy, Communication and Social Mobilization (ACSM), Information Education and Communication (IE&C) carried out behaviour change communication (BCC) activities during the year. The developed policy guidelines, manuals and a number of materials including data tools, printed and distributed to all regions [20]. Despite the high level of awareness about malaria mode of transmission (86%**)** and methods of prevention (85%**)**, most of the participants had no LLIN. However a study found that the combination of IRS and LLIN provided significantly greater protection than the protection provided by ITN alone in preventing *P. falciparum* infection [21].

Although mobile populations are generally described as hard-to-reach, this study used GPS technologies to identify a point of access to the target population where malaria control programmes could carry out interventions. Reports of malaria are increasing in many countries and in areas thought free of the disease. One of the factors contributing to the re-emergence of malaria is human migration. People move for a number of reasons, including environmental deterioration, economic necessity, conflicts, and natural disasters. These factors are most likely to affect the poor, many of whom live in the malaria-endemic countries. Identifying and understanding the influence of these population movements could improve prevention measures and malaria control programmes [8].

### Limitations

The study had limitation in the sampling method used to recruit participants. Consecutive method was used because hawkers and long-distance truck drivers are hard to reach and do not have a census.

## Conclusion

The prevalence of malaria is high among hawkers and long-distance truck drivers. The factors significantly associated with malaria in this population includes marital status, occupation and educational level. The GPS identifies the station locations associated with the prevalence of malaria in this mobile populations which will be useful in malaria interventions in Ghana.
